# Terrestrial biome distribution in the Late Neogene inferred from a black carbon record in the northeastern equatorial Pacific

**DOI:** 10.1038/srep32847

**Published:** 2016-09-08

**Authors:** Donghyun Kim, Yong Il Lee, Kiseong Hyeong, Chan Min Yoo

**Affiliations:** 1 School of Earth and Environmental Sciences, Seoul National University, Seoul 08826, Korea; 2 Deep-sea and Seabed Resources Research Division, Korea Institute of Ocean Science & Technology, Ansan, 15629, Korea

## Abstract

The appearance and expansion of C_4_ plants in the Late Cenozoic was a dramatic example of terrestrial ecological change. The fire hypothesis, which suggests fire as a major cause of C_4_ grassland is gaining support, yet a more detailed relationship between fire and vegetation-type change remains unresolved. We report the content and stable carbon isotope record of black carbon (BC) in a sediment core retrieved from the northeastern equatorial Pacific that covers the past 14.3 million years. The content record of BC suggests the development process of a flammable ecosystem. The stable carbon isotope record of BC reveals the existence of the Late Miocene C_4_ expansion, the ‘C_4_ maximum period of burned biomass’ during the Pliocene to Early Pleistocene, and the collapse of the C_4_ in the Late Pleistocene. Records showing the initial expansion of C_4_ plants after large fire support the role of fire as a destructive agent of C_3_-dominated forest, yet the weak relationships between fire and vegetation after initial expansion suggest that environmental advantages for C_4_ plants were necessary to maintain the development of C_4_ plants during the late Neogene. Among the various environmental factors, aridity is likely most influential in C_4_ expansion.

Various lines of evidence such as stable carbon isotope data^1^,^2^, phylogenetic structure^3^, fossil phytoliths^4^, and macrofossils^5^ show an abrupt expansion of C_4_ plants during the Late Neogene, yet the cause of this ecological event is controversial. Decreased atmospheric CO_2_ concentration^1^,^6^,^7^, climate drying^4^,^8^,^9^,^10^, and fire^11^,^12^ have been suggested as possible causes. Among these, the fire hypothesis is gaining support from African vegetation modeling^13^ and sharp increase in charcoal flux in ocean sediment cores from the North Pacific^14^ and Atlantic^15^,^16^ Oceans during the Late Miocene. These studies focused mainly on matching the timing of fire with the initial C_4_ grassland expansion event, but they did not provide further comparison between the two events.

Black carbon (BC), defined as a carbon continuum formed by incomplete combustion of fossil fuels and plant materials, includes many different carbon compounds such as soot, charcoal, and other pyrogenic carbons^17^. Soot has the lowest reactivity and a very long tracer range of up to 1000 s of km^18^, making it an important fraction of marine sedimentary organic carbon^19^,^20^. The BC in deep-sea sediment is not affected by coastal or shelf processes or other disturbances. It is assumed that BC of pre-industrial periods was formed mainly by fire events of natural vegetation and that the amount of BC can provide the scale of fire that occurred in the continents upwind. Additionally, the stable carbon isotope composition of BC preserves the characteristics of the original plant biomass^21^,^22^. Thus, the BC record in deep-sea sediments has the potential to provide continuous information about long-time interactions between fire and vegetation.

In this study, we use BC in deep-sea sediments retrieved from the northeastern equatorial Pacific Ocean (Fig. 1) as a proxy to reconstruct the terrestrial vegetation and fire history for the past 14.3 million years. First, we present the content of BC, which is related to the scale of fire events. Then, we present the carbon isotope composition of BC to reconstruct paleovegetation history. The studied BC record can be divided into two time intervals (14.3–7.6 Ma and post −7.6 Ma) with different source regions (the Southern Hemisphere and the Northern Hemisphere, respectively^23^,^24^,^25^,^26^), revealing interactions between fire and vegetation. With this information, it is possible to evaluate the role of fire in late Cenozoic C_4_ grassland development and expansion.

## Results

### BC content record

The content of BC in pre-industrial deep-sea sediments reflects both the quantity of burned biomass and the wind transport mechanism. The quantity of burned biomass is affected by several factors. The productivity of vegetation provides the potential supply of fuel, and aspects of the fire events, such as frequency, size, intensity, type, and severity^27^, determine the amount of BC produced. The type of vegetation burned would also affect BC production, because forest fire has a higher emission factor than savanna or grass fire^28^. However, the low age resolution and broad source areas of deep-sea sediment do not help constrain the relative influence of these factors.

After being produced, BC is transported from continent to ocean as aerosol. It is reported that natural fire events occur intensively in a season with dry conditions^29^ or frequent lightning^30^. Thus, if seasonal winds from continent to ocean during fire seasons strengthen, the content of BC would increase compared to those of other eolian sediments. This case was reported from the South China Sea^31^, where the intensification of the East Asian winter monsoon in the Pleistocene was recorded in the corresponding BC content. Our study area is far from continents and thus is relatively free from local, seasonal winds, such as the Santa Ana in western North America and the Asian Monsoon. Accordingly, we may assume that the effect of seasonal winds would be relatively insignificant.

Down-core BC content ranges from 0.00% to 0.23%, with an average of 0.05% (Dataset 1; Fig. 2A). Prior to 7.6 Ma, when the sediment was mainly delivered from Southern Hemisphere continents, Central and South America^23^,^24^,^32^, the BC content averages 0.04% (range of 0.00–0.12%). Two periods with high BC content occurring at 14.3–12.7 Ma and 11.1–10.2 Ma are noticeable, and the BC content decreases gradually with time from the high values. These two periods coincide with arid periods in tropical South America^33^ (Fig. 2B), which indicates a potential relationship between tropical South American aridity and an increase in the quantity of burned biomass, because the arid climate results in low fuel moisture content and easy spread of fire. The gradual decrease of BC content following a peak can be explained by shortage of fuel due to frequent fires.

After 7.6 Ma, the eolian components are interpreted to have been sourced mainly from the continents in Northern Hemisphere, East Asia or North America^34^,^35^,^36^. The BC content ranges from 0.01% to 0.23% with an average of 0.05%. The BC content decreases gradually from 6 Ma to 4 Ma, then increases from 4 Ma to present. As open-habitat grassland became ecologically dominant in both North America^37^ and China^38^ before the Middle Miocene and maintained its dominance till date, it seems that the abrupt BC content change related with the burning of large forest would not have occurred and affected the BC content significantly. Thus, it is reasonable to regard low fire activity as the main cause of the decreasing BC content around 4 Ma. A humid time interval from about 6 Ma to 3.8 Ma in Asia^39^ (Fig. 2C) supports this interpretation, as the humid conditions may have suppressed fire events and decreased the quantity of burned biomass. After 4 Ma, increasing BC content suggests development of a new flammable ecosystem continues to exist till date, with increasing aridity in Asia due to uplift of the Tibetan Plateau (*i.e.* refs 39 and 40). Unlike the Southern Hemispheric record showing an abrupt increase of BC content, the Northern Hemispheric record shows a gradual increase of BC content from 4 Ma. This suggests that the flammable ecosystem in Northern Hemisphere continents developed gradually, with an increase in fuel through time.

From the observation that the BC content increases during arid conditions of source area (14.3–12.7 Ma and 11.1–10.2 Ma, Central and South America) and decreases during humid conditions of source area (around 5 Ma, Asia), it seems that the long-term scale of natural fires (quantity of burned biomass) varies mainly with the degree of aridity. In addition, the remarkably high BC contents at 6.8 Ma, 4.4 Ma, and 2.2–2.1 Ma are notable. These may indicate short-term, large-scale fire events, although the exact causes of such events are not clear at present.

Down-core BC mass accumulation rate (MAR) ranges from 0.33 mg/cm^2^/1000 yrs to 47.47 mg/cm^2^/1000 yrs (Fig. 2D). A sharp increase of MAR across 7.6 Ma is noticeable (3.70 mg/cm^2^/1000 yrs to 9.48 mg/cm^2^/1000 yrs on average). This is likely due to the increasing linear sedimentation rate^23^,^24^. Considering the changes in eolian source from Southern Hemisphere to Northern Hemisphere, the large difference in BC MAR across 7.6 Ma can be accounted for by the larger amount of landmass in the Northern Hemisphere than in the Southern Hemisphere, which increased the average production amount of eolian sediments including BC.

### Carbon isotope record

The δ^13^C value of BC ranges between −29.3‰ and −20.5‰ (Dataset 2; Fig. 2E). The δ^13^C values of BC can be divided into five time intervals (Dataset 3). (1) From the beginning of the core record at 14.3 Ma to 10.5 Ma, BC shows depleted δ^13^C values (−26.5 ~ −29.3‰), with the most depleted value from 11 Ma to 10.5 Ma. (2) From 10.5 Ma to 7.6 Ma, δ^13^C values are generally increased (−24.7 ~ −27.1‰) compared with the previous interval. (3) From 7.6 Ma to 5.3 Ma, δ^13^C values are more increased than values of the immediately preceding interval. The amplitude of δ^13^C fluctuation is also increased visibly (−23.1 ~ −29.1‰). (4) The interval with the highest δ^13^C values (−20.5 ~ −28.6‰) is the interval from 5.3 Ma to 1.2 Ma. (5) After 1.2 Ma, the δ^13^C value decreases gradually, until reaching a value near −29‰ (−23.3 ~ −28.8‰).

By correcting the carbon isotope fractionation factor for the burning of vegetation and the isotopic composition of atmospheric CO_2_, the approximate composition of C_3_ and C_4_ vegetation of BC can be estimated. The five time intervals representing the carbon isotope composition variations can be understood in the context of vegetation development (Fig. 2F). From the beginning of the core record, 14.3 Ma to 10.5 Ma, C_3_ plants in Central and South America occupied nearly 100% of BC, and there is no isotopic evidence for the existence of C_4_ vegetation. The first recognizable presence of burned C_4_ plants occurs at 10 Ma, which is about 2 million years earlier than the timing of the major C_4_ expansion. This represents the existence of patches of C_4_ vegetation at that time, which agrees with the stable carbon isotope records of rodent teeth in Argentina^41^. After this time, the contribution of C_4_ plants to BC maintains a value up to 10%, indicating that C_4_ vegetation in Central and South America continued to exist from 10 Ma, although it did not become the major terrestrial vegetation.

After 7.6 Ma, the contribution of C_4_ plants reaches up to 20% of the burned biomass, suggesting the possible existence of open-canopy C_4_ grassland environment in the source areas. As 7.6 Ma is close to the timing of global expansion of C_4_ vegetation (~8 Ma), the estimation of C_4_ vegetation proportion using this method is consistent with other available information (*i.e.* ref. 3). The increasing contribution of C_4_ vegetation continued until reaching its highest proportion (up to 50%) of the BC in the fourth time interval, forming the ‘C_4_ maximum period of burned biomass’ during the Pliocene to Early Pleistocene in the Northern Hemisphere. The beginning of the C_4_ maximum proportion in the terrestrial ecosystem in the Northern Hemisphere seems largely coincident with the expansion of C_4_ vegetation in East Asia (Chinese Loess Plateau^42^ and central Inner Mongolia^43^, China). After 1.1 Ma, C_3_ plants gradually regained their dominance, reaching more than 80% of burned vegetation. The diminishment of C_4_ plants in the Late Pleistocene can be observed in other data from China^31^,^43^,^44^,^45^ and North America^4^ (Fig. 3).

## Discussion

The BC content represents the quantity of burned biomass, whereas the carbon isotope composition of BC provides information about the type of vegetation that was burned. The combination of these two parameters therefore indicates a linked relationship between fire activity and type of vegetation burned in the ecosystem.

The relationship between fire and vegetation in Central and South America can be explained easily. Large-scale fire events in South America (11.1–10.2 Ma) are associated with the most depleted δ^13^C values, indicative of large-scale forest burning, which was followed by, although minor, the first recognizable presence of C_4_ plants. A similar observation can be made from South China Sea data, in which a large-scale fire event near 3.5 Ma was followed by an abrupt increased contribution of C_4_ vegetation to burned vegetation^31^. These observations suggest that fire served as a trigger for early C_4_ expansion by burning C_3_ forests and eventually providing open habitats for C_4_ grasses which are more adaptable to an environment frequently prone to fire^46^.

The cases of South America (this study) and China^31^,^45^ both suggest C_4_ expansion events triggered by breakout of fire. However, the proportion changes of burned C_4_ vegetation after the fire event is clearly different. In South America, the C_4_ vegetation just formed patches in the ecosystem after the fire event from 11.1–10.2 Ma (0–10%), while the C_4_ vegetation in South China shows significantly increased contribution after the fire event from 4–3 Ma (20–40%, calculated by the method of this study). This variation in C_4_ development process between each case seems to be caused by the difference of circumstance. The South American case occurred prior to a steep decline in atmospheric CO_2_ that occurred at ~7 Ma^7^, whereas the Chinese event occurred after that. Thus, it is reasonable to guess that lower atmospheric CO_2_ provided conditions more favorable to C_4_ vegetation in the case of China, resulting in the more dramatic expansion of C_4_ vegetation.

After C_4_ vegetation occupied certain portion of the ecosystem, short-term large fire events (6.8 Ma, 4.4 Ma, and 2.2–2.1 Ma) do not show a clear correlation with a dramatic increase of C_4_ vegetation. Instead, when the contribution of C_4_ vegetation is over 20%, which indicates the existence of open-canopy condition, carbon isotope value and BC content show weak negative correlation (coefficient of correlation = 0.33). It shows that increased fire events occur with low carbon isotope value, which indicates C_3_ vegetation burning, whereas decreased BC production coincides with increased contribution of C_4_ vegetation. This relationship resembles, although minor, the fire-triggered C_4_ development event which can be characterized by high BC content–low δ^13^C value due to the burning of C_3_ vegetation followed by low BC content–high δ^13^C value which represents C_4_ vegetation development. Thus, the role of fire after the establishment of open-canopy environment seems to maintain C_4_-dominated environment, rather than causing a major event of C_4_ vegetation. Also, low coefficient of correlation between carbon isotope value and BC content suggests not only fire but also other environmental factors, such as CO_2_, temperature, seasonality, precipitation^8^,^47^,^48^, or edaphic ghettos^49^, would have played roles in controlling the C_3_–C_4_ ratio of the ecosystem, as shown from the case of modern vegetation^50^.

The effect of environmental factors rather than fire on the contribution of C_4_ vegetation to the ecosystem can be observed more clearly in the Late Pleistocene, when the long-term increase of BC content (4 Ma to present) co-exists with the latter gradual ceasing of C_4_ plant expansion (1.2 Ma to present). This event may have been affected by decreased sea-surface temperatures after the mid-Pleistocene transition^51^, which controlled the atmospheric moisture content and consequentially the aridity^52^, or the decreasing temperature conditions from the Middle Miocene onward^53^ may have reached a threshold value for C_4_ plant survival. The latter is more likely to be the main cause of decreasing C_4_ plants, as the known records of C_4_ decrease during Pliocene to Pleistocene are from high-latitude regions, and most records from low-latitude regions do not indicate such a decrease in C_4_ plants (Fig. 3).

To summarize, a drastic expansion of C_4_ vegetation triggered by fire breakout requires the following conditions: (1) an original landscape composed of closed habitat, so fire events can provide high light conditions for C_4_ grasses; (2) environmental conditions that are favorable to C_4_ vegetation, so C_4_ grasses can continue developing in competition with C_3_ grasses like lower atmospheric CO_2_; and (3) environmental conditions favorable to fire. After the open-canopy environment is established, the influence of fire as a driver of drastic ecosystem development wanes, yet fire still plays a role in maintaining C_4_-dominated ecosystem as one of the environmental conditions.

Considering these hypotheses, we suggest aridity as the most important factor that induced C_4_ expansion, as aridity can provide both opportunity for fire breakout and environmental conditions favorable to C_4_ vegetation growth and sustainment. Under general arid conditions, clearance of forest by fire events would result in changes to the hydrological cycle through reductions of evapotranspiration and cloud formation, thus enhancing the arid conditions. In North Africa, aridification of the Sahara Desert caused by the shrinkage of the Tethys Sea during the Late Miocene preceded the 8 Ma C_4_ expansion event^54^ and higher concentrations of charred particles^16^, suggesting that the aridification strengthened the fire activity and eventually led to C_4_ expansion. In South Asia, the 8 Ma C_4_ expansion event^8^ matches with the timing of Asian drying caused by the Tibetan uplift^39^, and the later C_4_ expansion event in East Asia seems to have been affected by the evolution and change in the monsoon climate that increased the seasonal aridity^31^,^55^,^56^. As the origination of C_4_ lineages is interpreted to have begun at 35 Ma and major diversification began at around 15 Ma^57^, aridity events after 15 Ma might have reasonably provided favorable environmental conditions coupled with open habitat formed by fire events, resulting in the expansion of C_4_ grassland. The long C_4_ maximum period of burned biomass from the Pliocene to the Late Pleistocene may reflect a time interval having increased territory with water stress favorable to C_4_ vegetation, until the low temperature effect surpassed the advantages provided by aridity. The latter effect resulted in the eventual C_4_ vegetation decrease of the Late Pleistocene.

## Methods

### Sample description

**Figure 1:**
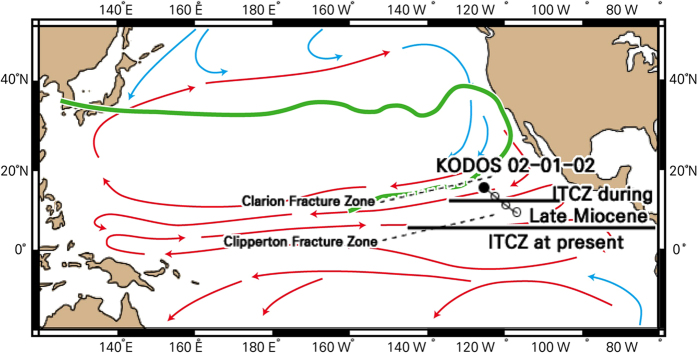
Location of core site KODOS 02-01-02. Open symbols represent the backtrack path of the study site with increments of 5 Myr. Paleo-locations of the ITCZ are shown as horizontal lines: for Late Miocene^23^ and for present^26^, respectively. Blue and red arrows represent modern main ocean currents. Green bold line shows the wind trajectory from East Asia to the study area (March 20, 1984)^36^. Modified by PaintTool SAI from Global Multi-Resolution Topography (GMRT) MapTool^71^.

The sediment samples used in this study are from a 328-cm-long piston core (KODOS 02-01-02) collected at 16°12′N, 125°59′W at a water depth of 4550 m in the Clarion-Clipperton fracture zone of the northeastern equatorial Pacific (Fig. 1). The chronology and mineralogical and geochemical characteristics of the studied core were reported by Hyeong *et al.*^23^,^24^ and are summarized briefly below. The coring site is located more than 2000 km from the East Pacific Rise and the nearest land mass (North and Central America) and thus is composed mostly of pelagic sediments. The core sediments consist of slightly bioturbated homogeneous siliceous red clays and are divided into two intervals based on a distinct color change; dark brown upper interval above 250 cm from the core top and yellowish brown lower interval below 250 cm. The inorganic fraction of the bulk pelagic sediments is considered to be eolian dust. The eolian sediment of the lower interval below 250 cm (15.5–7.6 Ma) is composed of smectite- and phillipsite-rich minerals with a very uniform mass flux of 5 ± 1 mg/cm^2^/10^3^ yr, whereas that in the upper interval above 250 cm (~7.6 Ma) is characterized by a quartz- and illite-rich mineralogy with a mass flux over 2 times higher than that of the lower interval at 12 ± 1 mg/cm^2^/10^3 ^yr. The eolian components in the lower interval are interpreted to have been sourced from Central and South America, whereas those in the upper interval are from Northern Hemisphere continents. It was interpreted that the distinctive differences in the eolian dust source between the lower and upper intervals of the studied core are due to a change in the paleolatitude of the Intertropical Convergence Zone which moved southwards through the coring site at ~7.6 Ma (Fig. 1).

### Analytical methods

In total, 189 samples collected at a 1 cm interval from the split core were used to determine BC concentration. There are four main techniques to determine the BC content of sediment: thermal, chemical, optical, and molecular marked^58^. Each of these procedures measures a different region within the combustion continuum^17^. Among these, the thermal and chemical methods are known to detect the highly condensed BC^18^. These two methods do not detect charcoal, but as charcoal is scarce in the deep-sea environment, the problem of limited detection of charcoal can be ignored. The thermal method produces artifacts by the charring of samples, which may result in overestimation of BC^59^. The chemical method using dichromate acid requires different mass yield corrections for each type of sample, yet it still allows precise, reproducible isotope measurements^60^. Because isotopic analysis was essential to reconstruct vegetation history, we followed the chemical oxidation method of Lim and Cachier^61^ for BC extraction.

Carbon content was obtained using an elemental analyzer (FlashEA 1112). The error range was ±3.3% of the reported value. By applying the total carbon value and weight of sample after chemical oxidation, the BC content in total sediment was calculated. Content of BC was analyzed using a CN analyzer (NA Series 2, CE Instruments, Italy). Carbon isotopic values of BC were analyzed by a stable isotope ratio mass spectrometer (IsoPrime-EA, Micromass, UK) interfaced with a CN analyzer at the National Instrumentation Center for Environmental Management, Seoul National University. Carbon isotopic values were presented in ‰ deviation from the Peedee belemnite (PDB). The analytical precision based on repeated measurements of the laboratory standard was better than ±0.1‰ (*i.e.* ref. 62).

### Converting δ^13^C value into distribution of vegetation type

To use stable carbon isotope data of BC as a proxy to reconstruct vegetation history, estimation of C_3_ and C_4_ plant δ^13^C composition was made by correcting the stable carbon isotope composition of paleoatmospheric CO_2_ in the studied time interval and the carbon isotope fractionation factor for vegetation burning. The isotope composition of paleoatmospheric CO_2_ has been obtained from contemporary marine carbonate. Following Ekart *et al.*^63^, we assumed that the difference in isotope composition between surface ocean carbonates and atmospheric carbon dioxide is 8‰ based on the isotope composition of pre-industrial carbon dioxide trapped in glacial ice^64^ and contemporaneous surface ocean carbonates^65^,^66^. A similar apparent isotope enrichment value, 7.9 ± 1.1‰, was suggested by Passey *et al.*^67^ using δ^13^C of planktonic foraminifera calcite tests as a proxy for δ^13^C of atmospheric CO_2_. The geologic record of Miocene–Pleistocene open ocean carbonates of Shackleton *et al.*^68^ was used to calculate δ^13^C of the paleoatmospheric carbon dioxide (δ^13^C_pCO2_) during the studied time interval, which can be calculated by subtracting 8‰ from δ^13^C of planktonic foraminifera (δ^13^C_FORAM_).

Laboratory burning of C_3_ and C_4_ vegetation has revealed that there exists significant fractionation of carbon isotopes between the parent material and the corresponding aerosol (soot) produced during burning^21^,^22^,^69^. Aerosol derived from burning of C_3_ vegetation is enriched in ^13^C by 0.5‰, whereas aerosol from burning of C_4_ vegetation is depleted in ^13^C by 3.5‰. The average δ^13^C values of modern C_3_ plant, −28.5‰^70^, and of modern C_4_ plant, −12.5‰^1^, are used as end-member values for estimation of the C_3_–C_4_ biomass ratios present in the ecosystem. Compared to C_4_ plant, the δ^13^C range of C_3_ plant is large, depending on the environmental conditions, and values lighter than −28.5‰ are considered to represent environments receiving mean annual precipitation higher than the threshold precipitation for sustaining C_3_ forest (1500 mm/year^70^). We assumed that the δ^13^C of BC produced from modern C_3_ plant burning (δ^13^C_mBC−C3_) is −28‰ and that the δ^13^C from modern C_4_ plant burning (δ^13^C_mBC-C4_) is −16‰.

From these assumptions, the approximate shifts in δ^13^C of burned C_3_ and C_4_ vegetation through the time interval can be estimated as follows, considering the difference between δ^13^C of modern atmospheric carbon dioxide (δ^13^C_mCO2_) and δ^13^C_pCO2_:


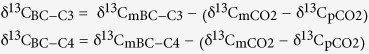


where δ^13^C_BC-C3_ and δ^13^C_BC-C4_ are the carbon isotope compositions of BC from C_3_ plant and BC from C_4_ plant, respectively. If we assume that δ^13^C_mCO2_ is about −8‰, and δ^13^C_pCO2_ can be substituted as (δ^13^C_FORAM_ −8‰), this fomular can be simplified like below:


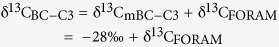



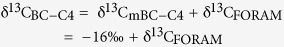


Using these values, the approximate composition of C_3_ and C_4_ vegetation burned can be estimated by the following simple mass-balance relationship:


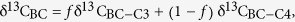


which can be changed as


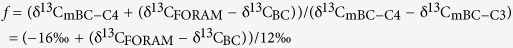


where *f* is the fraction of BC from C_3_ plant, (1−*f*) is the fraction of BC from C_4_ plant.

**Figure 2:**
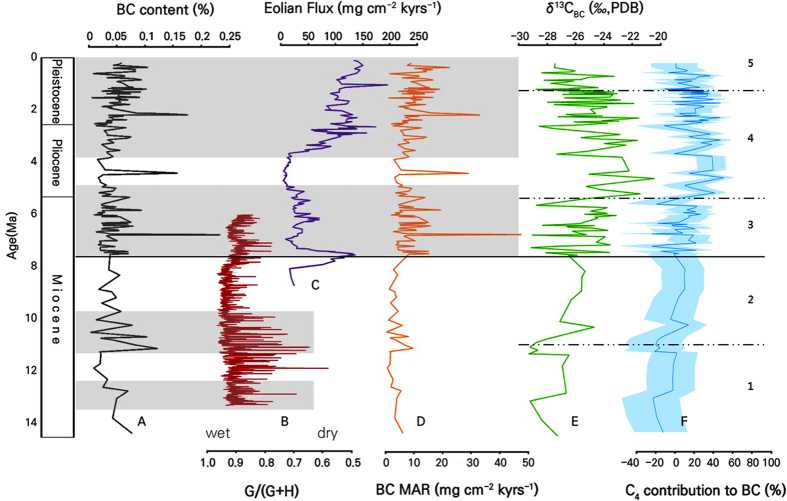
(**A**) BC content from deep-sea core sediment obtained from the northeastern equatorial Pacific. (**B**) Goethite/(goethite + hematite), G/(G + H) value at ODP Site 926 to represent aridity of Amazon and proto-Amazon lowland source areas. Lower G/(G + H) value suggests more arid condition (modified from Harris and Mix^33^). (**C**) Mass accumulation rate of eolian dust at ODP Site 885/886 (modified from Rea *et al.*^39^). (**D**) BC mass accumulation rate (MAR), (**E**) BC δ^13^C value, and (**F**) calculated proportion of C_4_ plants from deep-sea core sediment obtained from the northeastern equatorial Pacific. Black bold line across the center represents the time interval when the source area changed from Southern Hemisphere to Northern Hemisphere. Grey areas represent arid time intervals. Blue area represents error range assuming extreme environmental condition(closed-forest to steppe). Dotted lines in (**E**) and (**F**) define five time intervals (1–5) showing noticeable characteristics in δ^13^C value (see text for details).

**Figure 3:**
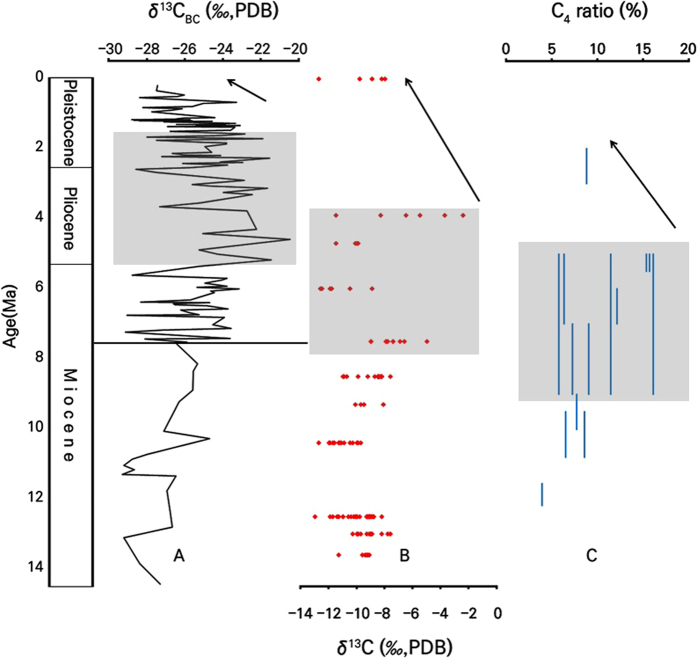
(**A**) δ^13^C value of BC from deep-sea core sediment obtained from the northeastern equatorial Pacific (this study). (**B**) δ^13^C value of tooth enamel form central Inner Mongolia, China (modified from Zhang *et al.*^43^). (**C**) Portion of C_4_ plant of fossil phytoliths from the Great Plains, North America (modified from Strömberg and McInerney^4^). Note the decrease of δ^13^C value or portion of C_4_ plant (black arrows) after the development of C_4_ ecosystem (grey areas).

In this calculation, we assumed δ^13^C_mBC−C3_ and δ^13^C_mBC−C4_ as constant value by fixing carbon isotope fractionation during combustion, and δ^13^C values of modern plants. However, δ^13^C value of BC varies by diverse factors under real conditions, which makes the f value as a rough estimate. δ^13^C value of C_3_ plant changes by the climate region. If we assume the whole C_3_ plants burned were from closed-forest canopy (δ^13^C = −32‰), f value will decrease <28% from the original value. If we assume the whole C_3_ plants burned were from semi-arid steppe environment (δ^13^C = −26%), f value will increase <30% (Fig. 2F). Also, both C_3_ (−0.6 to 1.8%) and C_4_ (−0.9 to −7.0%) vegetation has a certain range of carbon isotope fractionation during burning^22^, which increases a possible error range. This error range of *f* increases with (δ^13^C_FORAM_−δ^13^C_BC_) value. In our study, the maximum margin of error can be found at 7.2 Ma (δ^13^C_FORAM_−δ^13^C_BC_ = 30.90%), where *f* ranges from 0.91 to 2.43 with the average of 1.24, while the minimum margin of error can be found at 4.57 Ma (δ^13^C_FORAM_−δ^13^C_BC_ = 21.89%), where *f* ranges from 0.18 to 0.79 with the average of 0.49. On average (δ^13^C_FORAM_−δ^13^C_BC_ = 26.60%), *f* ranges from 0.91 to 2.43 with the average of 1.24.

## Additional Information

**How to cite this article**: Kim, D. *et al.* Terrestrial biome distribution in the Late Neogene inferred from a black carbon record in the northeastern equatorial Pacific. *Sci. Rep.* **6**, 32847; doi: 10.1038/srep32847 (2016).

## Supplementary Material

Supplementary Dataset 1

Supplementary Dataset 2

Supplementary Dataset 3
